# Ionic Liquid Solutions as a Green Tool for the Extraction and Isolation of Natural Products

**DOI:** 10.3390/molecules23071765

**Published:** 2018-07-18

**Authors:** Jiao Xiao, Gang Chen, Ning Li

**Affiliations:** School of Traditional Chinese Materia Medica, Shenyang Pharmaceutical University, Wenhua Road 103, Shenyang 110016, China; xj110121@126.com (J.X.); chengang1152001@163.com (G.C.)

**Keywords:** ionic liquids, bioactive natural products, herbals, extraction and isolation, flavonoids, alkaloids, terpenoids, phenylpropanoids

## Abstract

In the past few years, the application of ionic liquids (ILs) had attracted more attention of the researchers. Many studies focused on extracting active components from traditional herbals using ILs as alternative solvents so as to address the issue caused by the traditional methods for extraction of natural products (NPs) with organic chemical reagents. Through the summary of reported research work, an overview was presented for the application of ILs or IL-based materials in the extraction of NPs, including flavonoids, alkaloids, terpenoids, phenylpropanoids and so on. Here, we mainly describe the application of ILs to rich the extraction of critical bioactive constituents that were reported possessing multiple therapeutic effects or pharmacological activities, from medicinal plants. This review could shed some light on the wide use of ILs in the field of natural products chemistry to further reduce the environmental damage caused by large quantity of organic chemical reagents.

## 1. Introduction

Natural products (NPs) were the most important resource of leading compounds for drug discovery [1,2,3]. Indeed, approximately 40% of drugs available for clinical use were derived from natural chemical structures [4,5]. For example, the most famous antimalarial drug artemisinin, which was first isolated from *Artemisia annua* L., and the finding of it had been awarded the Nobel Prize in 2015 [6,7]. The reason for this success in drug discovery can probably be explained by the high chemical diversity of NPs across many species of the original plants [8]. While, discovery of the chemical diversity demands special methods to extract and isolate target products from different herbals [9]. Usually, organic solvent extraction was the optimal choice to obtain NPs. However, there were two major barriers for the discovery of NPs now. One was the low abundance of some active NPs in herbals [2,10]. In order to get enough amount of target products, large volumes of organic solvents were employed in the extraction and purification processes. The other barrier was the high repetition rate in the discovery of NPs across different species [11].

ILs were a group of organic salts, in general, composed of organic cation (e.g., imidazolium, pyrrolidinium, pyridinium tetraalkyl ammonium, tetraalkyl phosphonium) and inorganic or organic anion (e.g., tetrafluoroborate, hexafluorophosphate and bromide) and presented in the form of liquid when below 100 °C [12,13]. Now, ILs had been widely applied due to their unique and structure-dependent properties [14], such as the negligible vapor pressure [15], low nucleophilicity [16], miscibility with water or organic solvents, and good extractability for the organic compounds and metal ions. With these characteristics, ILs were more environmentally benign solvents comparing to the traditional volatile organic solvents used for extraction [17]. Therefore, many researchers had utilized IL-based technologies to extract and purify bioactive components from herbal medicines. Thus, this review would highlight the application of ILs in the extraction and purification of special bioactive NPs.

## 2. Extraction of Active Component Groups from Plants by ILs

Active component groups, such as flavonoids fraction, polysaccharides fraction and alkaloids fraction, had been reported as the active material basis of the traditional herbals. Herein, we summarized the application of ILs as a green tool for extraction flavonoids and polysaccharides fractions. All the acronyms of ILs described in this paper are listed in Table 1.

### 2.1. The Flavonoids Fraction

Flavonoids had been found as active ingredients in herbals, fruits, vegetables, tea, red wine and so on. Some of the compounds were well bioavailable after being ingested, and therefore exert diverse biological effects [18]. Here, we presented the reported application of ILs in the extraction of natural flavonoids fraction (Table 2), and described two research cases as follows.

The first case was the extraction of the flavonoids fraction from *Broussonetia papyrufera* (L.) Vent. It had been reported that flavonoids should be the major active composition of the traditional herbal medicine *B. papyrufera* (L.) Vent to treat dysentery, hypotension and cancer [19,20]. In the previous work, the flavonoids fraction was extracted by large amounts of volatile organic solvents including ethyl acetate, *n*-butyl alcohol, methanol, ethanol and so on, leading to inherent environmental concerns [21]. To overcome the major drawbacks of the traditional extraction methods, Wang and co-workers [21] employed [HBth][CH_3_SO_3_], a kind of functional IL, to extract the flavonoids fraction from leaves of the plant. As a result, the yield of the flavonoids fraction was doubled comparing with that of the traditional method without ionic liquids.

In the other case, Tan et al. reported [22] that the extraction of the flavonoids fraction from the leaves of *Apocynum venetum* [22,23,24,25] using the IL [C_4_mim][N(CN)_2_] aqueous solution. The extraction conditions of IL concentration, liquid/solid ratio, and ultrasonic time were optimized by RSM (response surface method), with further purification of the flavonoids by ABS (aqueous biphasic system). With the developed methodology, the yield of flavonoids reached 83.5%, increasing 32 folds compared with ultrasound-assisted extraction without ILs [26].

### 2.2. The Polysaccharides Fraction

Polysaccharides extensively existed in our dietary food, herbal medicines, and marine organism. Especially, many species of marine alga contained large quantities of polysaccharides with anti-tumor, anti-viral, anti-oxidant, anti-infective, and anti-mutagenic activities [41,42]. In Table 3, the application of ILs for extraction of polysaccharides fraction were listed.

Many species of marine alga, especially red and brown

Aloe polysaccharides were the major active constitutes of the *Aloe vera* L. [43]. Various methods were used for separation and purification of *Aloe* polysaccharides, such as ethanol precipitation, ion-exchange chromatography [44], and membrane separation [45]. However, Tan et al. [46] employed an IL ([Bmim]BF_4_) based aqueous two-phase system for the extraction of aloe polysaccharides. This modified method raised the yield of *Aloe* polysaccharides from 85.20% [44] to 93.12%.

## 3. Extraction of Single Compounds from Plants by ILs

The classical way of purification for single compounds from the plants started with extraction, and subsequently treated with repeated chromatography, such as macroporous resin, silica gel, Sephadex LH-20 column chromatography and HPLC methods [51]. These processes of purification were a waste of time and money [52]. However, ILs had been proposed to catch NPs [53] with their unique chemical functional groups [15]. Until now, there were 236 compounds characterized by ILs from plants reported in 167 references, including flavonoids (compounds **1**–**65**), alkaloids (compounds **66**–**129**), terpenoids (compounds **130**–**187**), phenylpropanoids (compounds **188**–**215**), quinones (compounds **216**–**233**), and others (compounds **234**–**236**). Their chemical structures are presented in Figures S1–S6.

### 3.1. Flavonoids (Compounds ***1***–***65***)

Flavonoids were built upon a C_6_–C_3_–C_6_ skeleton, in which the C_3_ unit usually characterized different types of flavonoids, including flavones, flavonols, isoflavones, chalcones, anthocyanidins and so on (Figure S1) [54]. Moreover, hydroxyl, methoxyl, and glycosyl groups commonly existed in flavonoid structures. All the flavonoids extracted from natural sources by the application of ILs were described in Figure S1 and Table 4. A total of 65 flavonoids were summarized here, including 41 flavones, 13 isoflavones, three flavanones and eight anthocyanidins.

Taking rutin (quercetin-3-*O*-rutinoside, compound **1**) as an example. It had been found in many herbs as the active component, including *Abutilon theophrasti* [54], *Tamarix Chinensis* [55], *Sophora japonica* L. and so on. It could be extracted by different solvent-extraction processes based on its acidity and polarity. For the polarity of rutin, ethanol-water was usually used to extract rutin under reflux [56]. While Ca(OH)_2_ solution was also employed to enrich acidic rutin [57]. However, the shortcomings of the traditional methods, such as low yield about 8% [56] and time-consuming restricted their further application. Hence, Zhao et al. [58] developed a new method using IL [C_4_mim]Br based on ultrasonic- and microwave-assisted extraction (UMAE) for extracting rutin from the leaves of *A. theophrasti*. The yield of rutin obtained by ILs-UMAE method was 5.49 mg/g, increasing 2.01 folds than that of heating reflux extraction with methanol. While, Wang [55] and co-workers used [Bmim]Br as solvent to extract rutin from *T. Chinensis*, and the yield of rutin was 0.0756%, which was 15.1% higher than that of the ultrasonic extraction with methanol.

### 3.2. Alkaloids (Compounds ***66***–***129***)

Alkaloids were a group of compounds containing one or more carbon rings and basic nitrogen atoms (Figure S2). [92]. According to their biosynthesis pathway and chemical characteristics, alkaloids were divided into phenylalanine-derived, tryptophan-derived, and terpenoid alkaloids, etc. In total 64 alkaloids extracted from natural sources by the application of ILs solutions were described in Figure S2 and Table 5.

Sinomenine (7,8-didehydro-4-hydroxy-3,7-dimethoxy-17-methylmorphinan-6-one, **66**), is a bioactive alkaloid isolated from the root and stem of *Sinomenium acutum* [93,94] and *Diploclisia affinis* [95]. There are two common ways to get sinomenine, one was alkalizing the plant material with Ca(OH)_2_ and NH_3_·H_2_O, then extracted them using benzene or chloroform [95,96], further separated it on macroporous resin. And the other was percolating or refluxing with 75% ethanol-water (*v*:*v*) [97,98] for 2 h. Li and co-workers [99] developed magnetic ionic liquids (MILs) ([C_2_OHmim] FeCl_4_) based on UAE to extract sinomenine from *S. acutum*. Compared with reflux extraction by 70% ethanol-water (*v*:*v*) and pure water, the yield of sinomenine with MILs increased 2.4 and 2.8 folds, respectively. In addition, ILs also reduced the extraction time course from 4 h to 30 min.

Dong [100] also employed an approach using ILs for extracting five alkaloids (compounds **85**–**89**) from *Physochlainae infundibularis*. Through investigating four critical factors on the yield of alkaloids, including concentrations of ionic liquid, the solid–liquid ratio, extraction time and temperature, Dong found that 0.05 mol/L [C_3_tr][PF_6_] aqueous solution had the highest extraction efficiency at 95.1%. It took less time (55 min as opposed to 80 min) for above ionic liquid aqueous solution compared to the extraction with water [101], to extract those five alkaloids from natural plants.

### 3.3. Terpenoids (Compounds ***130***–***187***)

The terpenoids were a large family of NPs with general formula of (C_5_H_8_)_n_, including monoterpene, sesquiterpene, diterpene, sesterpene, triterpene, and others [116,117] (Figure S3). All the terpenoids extracted from natural sources with ILs were described in Figure S3 and Table 6, which included a total of 58 terpenoids (19 monoterpenoids, nine sesquiterpenoids, 10 iridoids, two diterpenoids and 18 triterpenoids).

Paclitaxel (compound **169**), a well-known clinical antitumor drug, was a diterpenoid existing in various Taxus species [118,119,120,121]. Usually, there were two methods for the extraction of paclitaxel from the original plant. One was soaking with 95% ethanol-water for 16 h [122] at room temperature, and the other was refluxing with methanol [123,124]. Both of them required plenty of time for extraction. Therefore, Tan [125] used MILs [C_4_mim]FeCl_3_Br along with methanol solution for extracting paclitaxel from Taxus species. By optimizing major factors, such as IL amount, solid-liquid ratio, and ultrasonic time, the yield was improved to 0.224 mg/g, which was twice as much as that produced by traditional reflux extraction with methanol under reflux. Meanwhile, the main advantage of the proposed method versus the conventional one (soaking with 95% ethanol-water) was the considerable reduction of time from 16 h to 30 min.

### 3.4. Phenylpropanoids (Compounds ***188***–***215***)

The general skeleton of phenylpropanoids bore a series of C_6_–C_3_ units [141], for instance, coumarins and lignans were two important types of NPs. There were 28 phenylpropanoids extracted from natural sources using ILs as alternative liquid reagents (Figure S4 and Table 7).

Psoralen (compound **203**) was the critical active component in *Psoralea corylifolia* L. [142] and *Ficus carica* L. [143], usually extracted from the plant materials with ethanol-water [144,145] and subsequently purified by multiple chromatographic steps [146]. Wang and co-workers [143] optimized the method by transformation and purification of psoralen from *Ficus carica* L. leaves using pH-dependent IL ([Bmim]Br). The yield of psoralen obtained in this way was 30.21 mg/g, increasing 2.44-folds as opposed to that of conventional ethanol-citric acid method without ILs. Besides, in this example, there was no participation of organic solvent, such as methanol and ethanol in the process of extraction, making the proposed method more environmentally friendly.

Furthermore, Liu et al. [147] proposed a novel extraction process for the preparation of aesculin (compounds **207**) from *Cortex fraxini* using ionic liquid [C_4_mim]Br in 2015. Under the optimal conditions, the extraction efficiency of aesculin reached to 18.70 mg/g, which was 6-fold higher than that of the conventional technique refluxing with 75% ethanol-water [148].

### 3.5. Quinones (Compounds ***216***–***233***)

Quinones were a kind of compounds with a fully conjugated cyclic dione structure. Usually, they consisted of four types of skeletons: benzoquinone type, naphthoquinone type, phenanthraquinone type, and anthraquinone type. There have been 18 quinones extracted with ionic liquids in recent findings (Figure S5 and Table 8).

Tanshinone IIA (compound **224**) is one of the major components in the traditional Chinese medicine Danshen (*Radix Salvia miltiorrhiza*) [156]. Ethanol was usually used to extract tanshinone IIA under reflux [157]. However, the structure might be broken down because of the high temperature [158]. Hence, Liu and co-workers [159] selected 0.5 M IL (1-octyl-3-methylimidazolium hexafluorophosphate) in ethanol as solvent, to get tanshinone IIA from Danshen based on the ultrahigh pressure extraction. The yield of tanshinone IIA was 37.4 mg/g, which was 1.33-fold higher than that of the extraction method without ILs. Meanwhile, this approach potently cut the time from 120 min (with methanol) to 2 min (with ILs).

To explore mild and efficient techniques for separation and purification of aloe-emodin (compound **217**) from Aloe vera L., Tan et al. [160] evaluated the effects of the IL anion (Br^−^, BF_4_^−^ and N[CN]_2_^−^), the side chain length of cation alkyl in the [C_n_C_1_im]^+^ series, the extraction time, and the biomass-solvent ratio in the extraction of aloe-emodin. Under the optimal conditions, the extraction efficiency of aloe-emodin was 92.34%, increasing three folds compared with that of the heating reflux extraction with toluene [165]

### 3.6. Others (Compounds ***234***–***236***)

We also summarize three lactone compounds (compounds **234**–**236**, Figure S6 and Table 9) extracted with ILs. In order to improve the extraction process and develop a greener process, Chi et al. [166] investigated a MAE method to extract senkyunolide I (compound **234**), senkyunolide H (compound **235**) and Z-ligustilide (compound **236**) from *Ligusticum chuanxiong* Hort., using two ILs *N,N*-dimethyl-*N*-(2-hydroxyethoxyethyl) ammonium propionate (DMHEEAP) and *N,N*-dimethyl (cyanoethyl) ammonium propionate (DMCEAP) as solvents. The result showed that the MAE of the three target lactones using ILs took less time (within 5 min), instead of 74 min [167] extracted by high-pressure ultrasonic-assisted without IL.

## 4. Summary

In this review, we summarized 236 natural products isolated from plants with ILs based methods reported in 169 references. All the typical and important bioactive types of NPs were included, such as flavonoids (compounds **1**–**65**), alkaloids (compounds **66**–**129**), terpenoids (compounds **130**–**187**), phenylpropanoids (compounds **189**–**215**), quinones (compounds **216**–**233**) and others (compounds **234**–**236**). It was clear that ILs could be tailored for the extraction of special strutures. Depending on their unique properties, ILs present advantages in destroying the cell walls and capturing the targets by chemical selectivity so as to extract and efficiently enrich the products in a short time. However, the economic cost of some special ILs restricted their large scale of use for industry. In conclusion, ILs might serve as an accelerator for natural products chemistry research in the future.

## Figures and Tables

**Table 1:** Name and respective acronym of the cation-anion combinations in ILs.

Name	Acronym	Name	Acronym
benzothiazole	HBth^+^	methanesulfonic acid	CH_3_SO_3_^−^
1-alkyl-3-methylimidazolium	Cnmim^+^	dicyanamide	N(CN)_2_^−^
1-hydroxyethyl-3-methylimidazolium	C_2_OHmim^+^	tetrafluoroborate	BF_4_^−^
choline alanine	Ch^+^	bromide	Br^−^
1-butyl-3-methylimidazolium	Bmim^+^	tetrachloroferrate	FeCl4^-^
1-hexyl-3-methylimidazolium	Hmim^+^	bromotrichloroferrate	Fecl3Br^−^
1-octyl-3-methylimidazolium	Omim^+^	Leu	leucine^−^
2,2′-bipyridyl	BPy^+^	chloride	Cl^−^
3-methylimidazolium	Mim^+^	hexafluorophosphate	PF_6_^−^
1-(4-sulfonylbutyl)-3-methylimidazolium	HO_3_S(CH_2_)_4_mim^+^	hydrogenosulphate	HSO_4_^−^
1-ethyl-3-methylimidazolium	Emim^+^	acetic acid	OAc^−^
1-methylimidazolium-*p*-sulfonate	Pr mim^+^	bistrifluorosulfonimide	NTf_2_^−^
N-alkyl-hexafluorophosphate	Cntr^+^	L-aspartic acid	L-ASP^−^
tri-*n*-butylamine	TBA^+^	1-ethyl-3-methylimidazolium methylphosphonate	(MeO)(H)PO_2_^−^
1-butyl-3-methylimidazolium	CnCnim^+^	methoxyacetate	MOAc^−^
*N,N*-diethyl-*N*-methyl-*N*-(2-methoxyethyl)ammonium	DEME^+^	*p*-toluenesulfonic acid	p-TSA^−^
1-dodecyl-3-methylimidazole	Domim^+^		
1-methyl-3-(3-sulfopropyl)-imidazolium	PSmim^+^		
1-butyl-1-methylpyrrolidinium	C_4_C_1_pyr^+^		

**Table 2:** The application of ILs on extraction of the flavonoids fraction, and polysaccharides fraction.

Active Fraction	Types of ILs	Method of Extraction	Sources	References
flavonoids fraction	[HBth][CH_3_S0_3_]	MAE	*Broussonetia Papyrifera* (L.) L′ Hér. ex Vent.	[21]
	[C_4_mim][N(CN)_2_]	IL-UAE coupled with IL-ABS and back-extraction	*Apocynum venetum* L.	[25]
	[C_4_mim]Cl	aqueous two-phase system	*Phellinus igniarius* (L. ex Fr.) Quel	[27]
	[Bmim]Br	MAE	*Bauhinia Championii* (Benth.) Benth.	[28]
	[Bmim]Br	MAE	*Malus Micromalus* Makino	[29]
	[Ch][Leu]	maceration	*Citrus reticulata Blanco* cv. Ponkan	[30]
	[Bmim]BF_4_	UMAE	*Dendrobium nobile* L.	[31]
	[C_8_mim]BF_4_, [C_4_mim] BF_4_	aqueous two-phase system	*Ginkgo biloba* L.	[32]
	[Bmim]Cl	MAE	*Punica granatum* L.	[33]
	[Bmim]Br	MAE	*Smilax glabra* Roxb.	[34]
	[Bmim]Cl	aqueous two-phase system	*Cynoglossum zeylanicum (Vahl)* Thunb. ex Lehm.	[35]
	[Bmim]Br	MAE	*Pueraria lobata* (Willd.) Ohwi	[36]
	[Bmim]BF_4_	aqueous two-phase system	*Crataegus Pinnatifida* Bunge	[37]
	(Hmim)(PF_6_)	aqueous two-phase system	*Selaginella doederleinii* Hieron *trachyphylla* (*Warb.*) X.C.Zhang	[38]
	[C_4_MIM][PF_6_]	liquid−liquid extraction	*Crataegus Pinnatifida* Bunge	[39]
	[Bmim]Br	MAE	*Pueraria lobata* (Willd.) Ohwi	[40]

MAE: microwave-assisted extraction, UAE: ultrasound-assisted extraction, UMAE: ultrasound and microwave-assisted extraction, MPDE: multi-phase dispersive extraction, RSM: response surface method, IAE: infrared-assisted extraction.

**Table 3:** The application of ILs on extraction of polysaccharides fraction.

Active Fraction	Types of ILs	Method of Extraction	Sources	References
polysaccharide fraction	[Bmim]BF_4_	aqueous two-phase system	*Crataegus Pinnatifida* Bunge	[37]
	[Bmim]BF_4_	aqueous two-phase system	*Aloe vera* (Haw.) Berg	[46]
	1-(3-methoxypropyl)-3-methylimidazolium ethyl ethylphosphonate	liquid−liquid extraction	*Cryptomeria fortunei* Hooibrenk ex Otto et Dietr.	[47]
	SilprImNH_2_Cl	maceration	*Laminaria Japonica*	[48]
	[C_4_mim]BF_4_	UAE	*Zingiber officinale* Roscoe	[49]
	1-alkyl-3-methylimidazolium phosphonate and phosphinate-type ionic liquids	liquid−liquid extraction	*Boehmeria gracilis* C. H. Wright	[50]

**Table 4:** The flavonoids extracted from natural sources using ILs or IL solutions by different extraction techniques.

Compounds	Types of ILs	Methods of Extraction	Sources	References
rutin (**1**)	SilprBImCl, [Bmim]Br, [C_4_mim]Br, [Bmim]BF_4_[Hmim]Br, [Hmim]BF,	SPE, MAE, UMAE	*Artemisia capillaris* Thunb, *Saururus chinensis* (Lour.) Bail., *Xanthium sibiricum Patr., Tamarix Chinensis* Lour.	[54,55,56,59,60,61,62]
baicalein-7-O-glucoside (**24**), baicalein-7-O-diglucoside (**25**)	[C_4_mim][PF_6_]	aqueous two-phase system	*Veratrum stenophyllum* Diels	[59]
quercetin (**2**)	SilprBImCl, SilprImNH_2_, SilprPy, SilmPS, SiImBr, [C_4_mim]Br, [Bmim] Br, [Hmim] Br, [BPy]Br [HO_3_S(CH_2_)_4_mim]-HSO_4_	MDSPE, UMAE, MAE	*Artemisia capillaris* Thunb, *Xanthium sibiricum* Patr, *Toona sinensis* (A. Juss.) Roem., *Ginkgo bilobe* L.	[54,63,64,65]
kaempferol-3-*O-*β-D-glucoside (**5**)	Mim, [Bmim] Br, [Hmim] Br, [BPy]Br, [HO_3_S(CH_2_)_4_mim]HSO_4_	MAE	*Hippophae rhamnoides* L., *Toona sinensis* (A. Juss.) Roem, *Ginkgo bilobe* L.	[64,65]
isorhamnetin (**22**)	[HO_3_S(CH_2_)_4_mim]HSO_4_	MAE,	*Ginkgo biloba* L.	[65]
hesperidin (**3**), hyperoside (**4**)	[C_6_mim][BF_4_]	MAE	*Sorbus tianschanica* Rupr.	[66]
orientin (**6**), vitexin (**7**), genistin (**8**), isovitexin (**9**), luteolin-6-*C*-β-D-glucopyranosyl-8-*C*-*a*-L-arabinopyranoside (**10**), luteolin-6-*O*-α-L-arabinopyranosyl-8-*O*-α-D-glucopyranoside (**11**), apigenin-6,8-di-*O*-α-L-arabinopyranoside (**12**), apigenin-8-*O*-α-L-arabinoside (**13**)	[C_4_MIM]Br, [C_6_MIM]Br, [C_8_mim]Br	MAE, cavitation-assisted extraction	*Cajanus cajan* (L.) Millsp., *Glycine max* (L.) Merr.	[67,68,69]
ononin (**14**), daidzin (**15**), biochanin A (**16**), formononetin (**17**), puerarin (**18**), genistein (**19**), daidzein (**20**)	BMImBF_4,_ 1-butyl-3-methylimidazolium bromide, [Bmim][PF_6_], [Hmim][PF_6_], [Omim][PF_6_], [Bmim][NTf_2_], [C_6_min]Br, [C_8_mim]Br	MAE, liquid−liquid extraction, UAE, cavitation-assisted extraction	*Pueraria lobata* (Willd.) Ohwi, *Glycine max* (L.) Merr., *Cajanus cajan* (L.) Millsp.	[68,69,70,71,72]
glycitein (**42**)	[BMIm][PF_6_], [HMIm][PF_6_], [OMIm][PF_6_], [BMIm][NTf_2_],	liquid−liquid extraction	*Glycine max* (L.) Merr.	[72]
fisetin (**21**), kaempferide (**23**)	[C_4_mim][PF_6_], [C_6_mim][PF_6_], [C_8_mim][PF_6_], 1-butyl-3-methylimidazolium bromide	three phase micro extraction, MAE	*Rheum officinale* Baill., *Coptis chinensis* Franch., *Pueraria lobata* (Willd.) Ohwi	[71,73]
pelargonin (**26**), cyanidin (**27**), peonidin (**28**), petunidin (**29**), malvidin (**30**)	[C_2_mim]OAc	aqueous two-phase systems	*Vitis vinifera* L.	[74]
luteolin (**31**), apigenin (**32**)	[Bmim][MS], [C_12_mim]Br, [C_8_mim]Br	UAE, cavitation-assisted extraction	*Apium graveliens* L., *Chrysanthemum morifolium* Ramat, *Cajanus cajan* L. Millsp	[58,69,75]
kaempferide (**53**), acacetin (**54**)	[C_12_mim]Br	UAE	*Chrysanthemum morifolium* Ramat.	[75]
tectorigenin (**33**), iristectorigenin A (**34**), irigenin (**35**), irisflorentin (**36**)	[Emim][BF_4_]	UAE	*Belamcanda chinensis* (L.) Redouté	[76]
myricetin (**37**), amentoflavone (**38**)	1-oxyl-3-methylimidazolium bromide, [Emim][Cl], [Bmim][Cl], [Hmim][Cl], [Omim][Cl], [Dmim]Br, 1-bromodecane, 1-vinylimidazole	MPDE, SPE, UAE, SPE	*Chamaecyparis obtuse* (Sieb.et Zucc) Endl	[77,78,79,80]
dihydrokaempferol (**60**)	[Dmim]Br	UAE	*Chamaecyparis obtuse* (Sieb.et Zucc) Endl	[79]
catechin (**39**), epigallocatechin (**40**), epigallocatechin gallate (**41**)	[C_2_mim][Br]	heat extraction	*Elaeis guineensis* Jacq.	[81]
rhodiosin (**43**), rhodionin (**44**), herbacetin (**45**)	[BMIM]Cl, EMIMB	aqueous two-phase systems, UAE	*Rhodiola rosea* L.	[82,83]
baicalin (**46**), wogonoside (**47**)	[C_4_mim][PF_6_]	aqueous two-phase systems	*Scutellaria baicalensis* Georgi	[84]
quercitrin (**48**)	molecularly imprinted anion-functionalized poly(ionic liquid)s, [Emim][Cl], [Bmim][Cl], [Hmim][Cl], [Omim][Cl], [DMIM]Br, 1-bromodecane, 1-vinylimidazole	MPDE, SPE	*Chamaecyparis obtuse* (Sieb.et Zucc)Endl	[78,79,80,85]
herbacetin-3-O-β-d-glucopyranosyl-7-O-α-l-rhamnopyranoside (**49**), kaempferol-3-O-β-d-glucopyranosyl-7-O-α-l-rhamn-opyranoside (**50**), kaempferol 3-O-β-d-glucopyranoside-(2→1)-α-d-xylopyranoside (**51**), herbacetin-8*-O-β-d*–glucopyranoside (**52**)	1-ethyl-3-methylimidazoliumbromide	SPE	*Rhodiola rosea* L.	[86]
baicalein (**55**), wogonin (**56**)	[C_8_mim]Br	UAE	*Scutellaria baicalensis* Georgi.	[87]
procyanidin(**57**), anthocyanin-3-O-acetylmonoglucosides (**58**), anthocyanin-3-(6-O-p-coumaroyl)monoglucosides (**59**)	[C_4_mim][Br], [C_2_mim][Br], [mim][HSO_4_], [sC_4_mim][HSO_4_]	liquid−liquid extraction	*Vitis vinifera* L.	[88]
neohesperidin (**61**), naringin (**62**)	[Bmim]BF_4_, [Ch][Bic]	SPE, liquid−liquid extraction	*Vaccinium* Spp	[89]
hyperin (**63**)	[C_4_mim]BF_4_	MAE	*Populus euphratica* Oliv.	[90]
kaempferol-3,4′-di-O-β-D-glucoside (**64**), kaempferol-3-O-β-D-(2-O-β-D-glucosyl)-glucopyranoside (**65**)	[E mim][Cl], [Pr mim ][Cl], [B mim][Cl], [C_5_ mim][Cl], [E mim ][Br], [Pr mim][Br], [B mim][Br], [C_5_ mim ][Br], [EMIM][BF_4_], [Pr mim][BF_4_], [B mim ][BF_4_] and [C_5_mim][BF_4_]	aqueous two-phase systems	*Brassica napus* L.	[91]

**Table 5:** The alkaloids extracted from natural sources using ILs or IL solutions by different extraction techniques.

Compounds	Types of ILs	Methods of Extraction	Sources	References
sinomenine (**66**)	[C_2_OHmim]FeCl_4_	UAE	*Adenia chevalieri* Gagnep	[99]
anisodamine (**85**), atropine (**86**), scopolamine (**87**), aposcopolamine (**88**), scopoline (**89**)	[C_3_tr][PF_6_]	maceration	*Physochlainae infundibularis* Kuang (Solanaceae)	[100]
protopine (**67**), allocryptopine (**68**), sanguinarine (**69**), chelerythrine (**70**), dihydrochelerythrine (**71**), dihydrosanguinarine (**72**)	[C_6_mim][BF_4_], [K][PF_6_], 1-butyl-3-methylimidazole tetrafluoroborate	UAE, IAE	*Macleaya cordata* (Willd.) R. Br., *Dicranostigma leptopodum* (Maxim.) Fedde	[102,103]
quinine (**96**), cinchonidine (**97**), lycorine chloride (**112**)	[C_4_MIM]Cl	IAE	(Howard) Moens ex Trim.	[103]
aconitine (**73**), mesaconitine (**74**), hypaconitine (**75**)	1-ethyl-3-methylimidazolium tetrafluoroborate	UAE	*Aconitum carmic-haeli* Debx.	[104]
coptisine (**76**), palmatine (**77**), berberine (**78**), boldine (**79**), chelidonine (**80**), papaverine (**81**), emetine (**82**), columbamine (**83**), magnoflorine (**84**)	1-butyl-3-methylimidazolium Tetrafluoroborate, 1-butyl-3-methylimidazole tetrafluoroborate, 1-butyl-3-methylimidazolium bromide	IAE, UAE	*Coptis chinensis* Franch., *Dicranostigma leptopodum* (Maxim.) Fedde, *Phellodendron amurense* Rupr.	[99,105]
jatrorrhizine (**123**)	1-butyl-3-methylimidazolium bromide	UAE	*Phellodendron amurense Rupr*	[105]
theobromine (**90**), theophylline (**91**)	1-methylimidazole based IL	SPE	*Camellia sinensis* (L.) O. Ktze.	[106]
pronuciferine (**92**), N-nornuciferine (**93**), nuciferine (**94**), roemerine (**95**)	[C_4_mim][BF_4_]	aqueous two-phase system	*Nelumbo nucifera* Gaertn	[107]
galantamine (**98**), narwedine (**99**), N-desmethylgalantamine (**100**), ungiminorine (**101**)	[C_4_C_1_im]Cl	SPE	*Leucojum aestivum* L. (Amaryllidaceae)	[108]
brucine (**102**), cinchonine (**103**), glaucine (**104**), codeine (**105**), laudanosine (**106**), noscapine (**107**)	1-butyl-2,3-dimethylimidazolium tetrafluoroborate, [C_4_C_1_im][Ace]	IAE, maceration	*Glaucium flavum* Cr.	[103,109]
liensinine (**108**), isoliensinine (**109**), romerine (**110**), neferine (**111**)	[C_4_mim][PF_6_]	aqueous two-phase systems	*Nelumbo nucifera* Gaertn	[110]
rhynchophylline (**113**), pteropodine (**114**), isomitraphylline (**115**), isopteropodine (**116**)	Bmim	MAE	*Ranunculus ternatus* Thunb.	[111]
benzoylmesaconine (**117**), benzoylaconine (**118**), benzoylhypaconine (**119**), mesaconitine (**120**), hypaconitine (**121**), aconitine (**122**)	[C_6_mim]Br	aqueous two-phase system	*Aconitum carmichaeli* Debx	[112]
matrine (**124**), oxymatrine (**125**)	silica-confined ionic liquids	SPE	*Sophora flavescens* Ait	[113]
vinblastine (**126**), catharanthine (**127**), vindoline (**128**)	[Amim]Br	UAE	*Catharanthus roseus*	[114]
bilobalide (**129**)	[C_4_mim]Cl	maceration	*Ginkgo biloba* L.	[115]

**Table 6:** The terpenoids extracted from natural sources using ILs or IL solutions by different extraction techniques.

Compounds	Types of ILs	Methods of Extraction	Sources	References
paclitaxel (**169**)	MIL [C_4_mim]FeCl_3_Br	UAE	*Taxus chinensis* (Pilger) Rehd.	[125]
α-pinene (**130**), 1,8-cineole (**131**), linalool (**132**), terpinen-4-ol (**133**), Borneol (**134**), α-terpineol (**135**), bornyl acetate (**136**), cubebene (**149**), α-copaene (**150**), bergamotene (**151**), β-caryophyllene (**152**), humulene (**153**), calamenene (**154**), cadina-1,4-diene (**155**)	Benzalconium lactate, Didecyldimethyl lactate, Benzalconium nitrate, Didecyldimetthyl nitrate, Tris(2-hydroxyethyl)methylamonium methylsulfate	UAE	*Cinnamomum cassia* Presl	[126]
scroside B (**137**), hemiphroside A (**138**), scroside A (**139**), scroside C (**140**), scroside D (**141**), scroside I (**142**), picroside (**158**), picroside III (**159**), picroside I (**160**), picroside II (**161**), specioside (**162**), 6-O-E-feruloyl catalpol (**163**), 2-O-β-D-glucopyranosyl-3,16,20, 25-tetrahydroxy-9-methyl-19-norlanosta-5,23-dien-22-one (**170**), 2-O-β-D-glucopyranosyl-3,16,20-trihydroxy-25-acetoxy-9-methyl-19-norlanosta-5,23-dien-22-one (**171**)	[BMIM][BF_4_]	UAE	*Picrorhiza scrophulariiflora* Pennell	[127]
ionone (**143**), linalool oxide pyranoid (**144**), linalool oxide furanoid (**145**)	[C_2_mim][(MeO)(H)PO_2_)]	maceration	*Osmanthus fragrans* (Thunb.) Lour. Cv. Aurantiacus	[128]
geranial (**146**), neral (**147**), geraniol (**148**)	[C_4_mim]Cl [C_2_mim][(MeO)(H)PO_2_)], [C_2_mim][(MeO)(H)PO_2_], [DEME]Cl, [DEME][MOAc].	maceration	*Cymbopogon citratus* (D. C.) Stapf, *Backhousia citriodora*	[129,130]
cynaropicrin (**156**)	1-alkyl-3-methylimidazolium chloride	liquid-liquid extraction	*Cynara, cardunculus* L.	[131]
paeoniflorin (**157**)	[Bmim]Br	MAE	*Paeonia suffruticosa* Andr	[132]
forskolin (**168**)	tetramethyl guanidium lactate	UAE	*Coleus forskohlii* (Willd.) Briq	[133]
ganoderic-acid ∑ (**172**)	[C_4_mim]Cl [C_2_mim][(MeO)(H)PO_2_)]	UAE	*Ganoderma lucidum* (Leyss. Ex Fr.) Karst.	[134]
ursolic (**173**), ursolic (**174**), betulinic acids (**175**)	[C_4_C_1_im][N(CN)_2_], [C_4_C_1_im][TOS], [C_4_C_1_im][SCN], [C_4_C_1_im][C_2_H_5_SO_4_], [C_4_C_1_py][N(CN)_2_], and [C_4_C_1_pyr]Cl,	liquid-liquid extraction	*Malus pumila* Mill.	[135]
3-oxotirucalla-7,24Z-dien-27-oic acid (**176**), 3α-hydroxytirucalla-7,24Z-dien-27-oic acid (**177**), 3α-acetoxytirucalla-7,24Z-dien-27-oic acid (**178**)	BmimBr	MAE	*Schinus terebinthifolius* Raddi	[136]
dongnoside E (**179**)	ChCl	deep eutectic solvents extraction	*Agave americana* L.	[137]
insenoside Rg1 (**180**), ginsenoside Re (**181**), ginsenoside Rf (**182**), ginsenoside Rb1 (**183**), ginsenoside Rc (**184**), ginsenoside Rb2 (**185**), ginsenoside Rd (**186**)	[C_3_mim]Br	UAE	*Panax ginseng* C. A. Meyer	[138]
sgenin (**187**)	[PSmim]HSO_4_	UAE	*Dioscorea opposita* Thunb.	[139]
morroniside (**164**), sweroside (**165**), loganin (**166**), cornuside (**167**)	[Domim]HSO4	maceration	*Cornus officinalis* Sieb. et Zucc.	[140]

**Table 7:** The phenylpropanoids extracted from natural sources using ILs or IL solutions by different extraction techniques.

Compounds	Types of ILs	Methods of Extraction	Sources	References
schisandrin (**188**), schisantherin (**189**), deoxyschisandrin (**190**), γ-schisandrin (**191**)	Bmim-BF_4_	aqueous two-phase system	*Schisandra chinensis* (Turcz.) Baill.	[143]
psoralen (**203**)	[Bmim]Br	liquid-liquid extraction	*Ficus carica* L.	[145]
schisandrol B (**192**), schisanhenol (**193**), deoxyshisandrin (**194**), schisandrin C (**195**)	[C_4_mim][BF_4_]	UAE	*Schisandra chinensis* (Turcz.) Baill.	[149]
rosmarinic acid (**196**), sodium danshensu (**197**), lithospermic acid (**198**), salvianolic acid B (**199**)	SiO_2_^.^Im^+.^PF_6_	SPE	*Salvia miltiorrhiza* Bunge	[150]
schizandrin (**200**), schisantherin A (**201**), γ-schizandrin (**202**)	[C_4_mim]Ac	maceration	*Schisandra chinensis* (Turcz.) Baill.	[151]
magnolol (**204**), honokiol (**205**)	[Bmim][BF_4_]	UAE	*Magnolia officinalis* Rehd. et Wils.	[152]
podophyllotoxin (**206**)	[Amim][BF_4_]	MAE	*Dysosma versipellis* (Hance) M. Cheng ex Ying, *Sinopodophyllum hexandrum* (Royle) Ying	[153]
aesculin (**207**), aesculetin (**208**)	[C_4_mim]Br	ultrasound-microwave synergistic extraction	*Fraxinus chinensis* Roxb	[147,154]
isopsoralen (**209**), bergapten (**210**), isobergapten (**211**), oxypeucedanin (**212**), imperatorin (**213**), osthole (**214**), isoimperatorin (**215**)	[C_6_mim][PF_6_]	liquid–liquid micro extraction	*Angelica dahurica* (Fisch. ex Hoffm.) Benth. et Hook. f. ex Franch. et Sav.	[155]

**Table 8:** The quinones extracted from natural sources using ILs or IL solutions by different extraction techniques.

Compounds	Types of ILs	Methods of Extraction	Sources	References
rhein (**225**), danthron (**226**), emodin (**227**), chrysophanol (**228**)	[Bmim]Cl, [Bmim]Br, [Bmim]BF_4_	UAE, MAE	*Rheum officinale* Baill.	[155]
dihydrotanshinone (**231**), miltirone (**232**)	1-octyl-3-methylimidazolium hexafluoro-phosphate	ultrahigh pressure extraction	*Salvia miltiorrhiza* Bunge	[159]
emodin (**216**), aloe-emodin (**217**), rhein (**218**), aloin (**221**)	[C_4_mim]BF_4_	liquid–liquid extraction	*Aloe vera* L.	[160]
tashinone IIA (**224**)	(3-aminopropyl)trimethoxysilane, 1-octyl-3-methylimidazolium hexafluoro-phosphate	imprinted functionalized ionic liquid-modified silica, ultrahigh pressure extraction	*Salvia miltiorrhiza* Bunge	[159,161]
cryptotanshinone (**222**), tanshinone I (**223**)	(3-aminopropyl)trimethoxysilane	imprinted functionalized ionic liquid-modified silica	*Salvia miltiorrhiza* Bunge	[161]
chrysophanol (**219**), physcion (**221**)	1-hexyl-3-methylimidazolium hexafluorophosphate	liquid–liquid micro extraction	*Rheum officinale* Baill	[162]
shikonin (**229**), β,β′-dimethylacrylshikonin (**230**)	[C_6_mim][PF_6_]	UMAE	*Lithospermum erythrorhizon* Sieb. et Zucc.	[163]
stibene glycoside (**233**)	[Bth][Br], [HBth][*p*-TSA]	liquid–liquid extraction	*Fallopia multiflora* (Thunb.) Harald.	[164]

**Table 9:** The other compounds extracted from natural sources using ILs or IL solutions by different extraction techniques.

Compounds	Types of ILs	Methods of Extraction	Sources	References
senkyunolide I (compound **234**), senkyunolide H (compound **235**), Z-ligustilide (compound **236**)	*N,N*-dimethyl-*N*-(2-hydroxyethoxyethyl)ammonium propionate, *N,N*-dimethyl(cyanoethyl)ammonium propionate	MAE	*Ligusticum chuanxiong* Hort.	[166]

## Supplementary Materials

The following are available online at http://www.mdpi.com/1420-3049/23/7/1765/s1, Figure S1: The structures of flavonoids extracted from natural sources using ILs or IL solutions. Figure S2: The structures of alkaloids extracted from natural sources using ILs or IL solutions. Figure S3: The structures of terpenoids extracted from natural sources using ILs or IL solutions. Figure S4: The structures of phenylpropanoids extracted from natural sources using ILs or IL solutions. Figure S5: The structures of quinones extracted from natural sources using ILs or IL solutions. Figure S6: The structures of other compounds extracted from natural sources using ILs or IL solutions.
