# Supplementary material for: Ionic Liquid Solutions as a Green Tool for the Extraction and Isolation of Natural Products
Source: Molecules. 2018 Jul 18;23(7):1765. doi: 10.3390/molecules23071765 (PMC6100307; doi:10.3390/molecules23071765)
Supplement: Supplementary file 1 [file molecules-23-01765-s001.pdf]

molecules

MDPI

Review

Ionic Liquid Solutions as a Green Tool for the Extraction and Isolation of Natural Products

Jiao Xiao, Gang Chen and Ning Li *

School of Traditional Chinese Materia Medica, Shenyang Pharmaceutical University, Wenhua Road 103, Shenyang 110016, China; xj110121@126.com (J.X.); chengang1152001@163.com (G.C.)
* Correspondence: liningsypharm@163.com; Tel.: +86-24-2398-6475; Fax: +86-24-3150-9368

Received: 11 June 2018; Accepted: 14 July 2018; Published: date

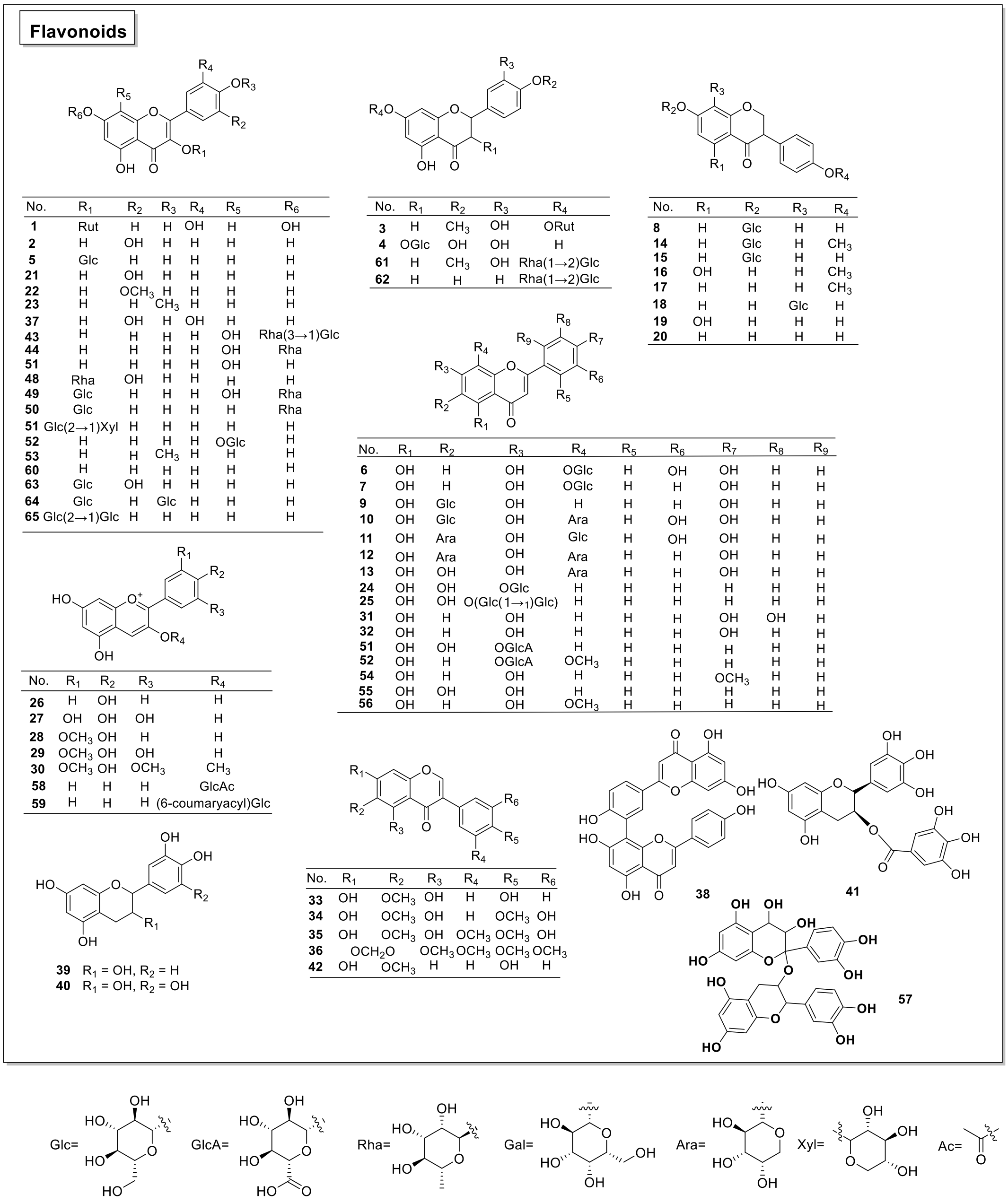

No.	R_1	R_2	R_3	R_4	R_5	R_6
**1**	Rut	H	H	OH	H	OH
**2**	H	OH	H	H	H	H
**5**	Glc	H	H	H	H	H
**21**	H	OH	H	H	H	H
**22**	H	OCH_3	H	H	H	H
**23**	H	H	CH_3	H	H	H
**37**	H	OH	H	OH	H	H
**43**	H	H	H	H	OH	Rha(3→1)Glc
**44**	H	H	H	H	OH	Rha
**51**	H	H	H	H	OH	H
**48**	Rha	OH	H	H	H	H
**49**	Glc	H	H	H	OH	Rha
**50**	Glc	H	H	H	H	Rha
**51**	Glc(2→1)Xyl	H	H	H	H	H
**52**	H	H	H	H	OGlc	H
**53**	H	H	CH_3	H	H	H
**60**	H	H	H	H	H	H
**63**	Glc	OH	H	H	H	H
**64**	Glc	H	Glc	H	H	H
**65**	Glc(2→1)Glc	H	H	H	H	H

No.	R_1	R_2	R_3	R_4
**3**	H	CH_3	OH	ORut
**4**	OGlc	OH	OH	H
**61**	H	CH_3	OH	Rha(1→2)Glc
**62**	H	H	H	Rha(1→2)Glc

No.	R_1	R_2	R_3	R_4
**8**	H	Glc	H	H
**14**	H	Glc	H	CH_3
**15**	H	Glc	H	H
**16**	OH	H	H	CH_3
**17**	H	H	H	CH_3
**18**	H	H	Glc	H
**19**	OH	H	H	H
**20**	H	H	H	H

No.	R_1	R_2	R_3	R_4	R_5	R_6	R_7	R_8	R_9
**6**	OH	H	OH	OGlc	H	OH	OH	H	H
**7**	OH	H	OH	OGlc	H	H	OH	H	H
**9**	OH	Glc	OH	H	H	H	OH	H	H
**10**	OH	Glc	OH	Ara	H	OH	OH	H	H
**11**	OH	Ara	OH	Glc	H	OH	OH	H	H
**12**	OH	Ara	OH	Ara	H	H	OH	H	H
**13**	OH	OH	OH	Ara	H	H	OH	H	H
**24**	OH	OH	OGlc	H	H	H	H	H	H
**25**	OH	OH	O(Glc(1→1)Glc)	H	H	H	H	H	H
**31**	OH	H	OH	H	H	H	OH	OH	H
**32**	OH	H	OH	H	H	H	OH	H	H
**51**	OH	OH	OGlcA	H	H	H	H	H	H
**52**	OH	H	OGlcA	OCH_3	H	H	H	H	H
**54**	OH	H	OH	H	H	H	OCH_3	H	H
**55**	OH	OH	OH	H	H	H	H	H	H
**56**	OH	H	OH	OCH_3	H	H	H	H	H

No.	R_1	R_2	R_3	R_4
**26**	H	OH	H	H
**27**	OH	OH	OH	H
**28**	OCH_3	OH	H	H
**29**	OCH_3	OH	OH	H
**30**	OCH_3	OH	OCH_3	CH_3
**58**	H	H	H	GlcAc
**59**	H	H	H	(6-coumaryacyl)Glc

No.	R_1	R_2	R_3	R_4	R_5	R_6
**33**	OH	OCH_3	OH	H	OH	H
**34**	OH	OCH_3	OH	H	OCH_3	OH
**35**	OH	OCH_3	OH	OCH_3	OCH_3	OH
**36**	OCH_2O		OCH_3	OCH_3	OCH_3	OCH_3
**42**	OH	OCH_3	H	H	OH	H

Figure S1. The structures of flavonoids extracted from natural sources using ILs or IL solutions.

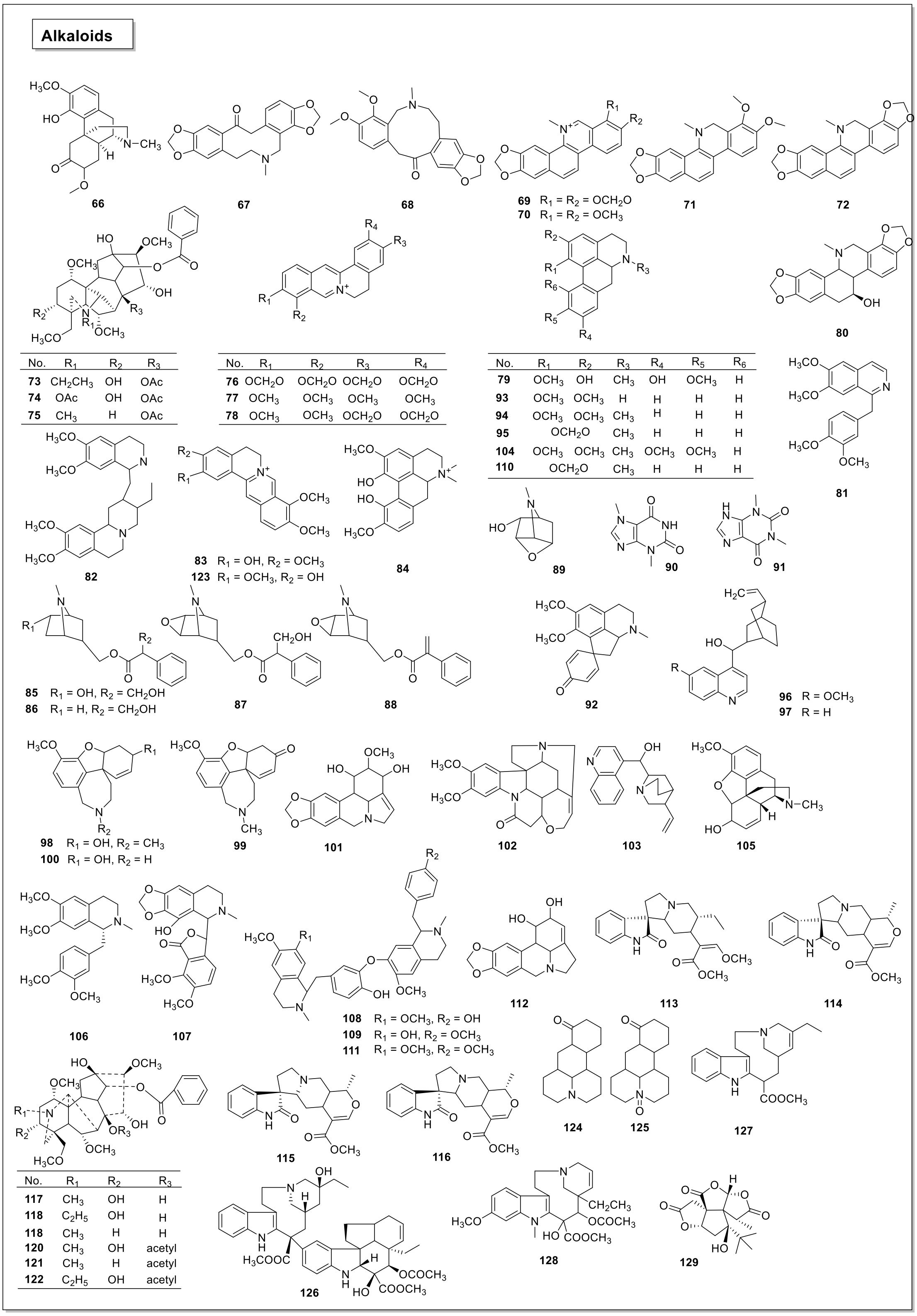

No.	R_1	R_2	R_3
**73**	CH_2CH_3	OH	OAc
**74**	OAc	OH	OAc
**75**	CH_3	H	OAc

No.	R_1	R_2	R_3	R_4
**76**	OCH_2O	OCH_2O	OCH_2O	OCH_2O
**77**	OCH_3	OCH_3	OCH_3	OCH_3
**78**	OCH_3	OCH_3	OCH_2O	OCH_2O

No.	R_1	R_2	R_3	R_4	R_5	R_6
**79**	OCH_3	OH	CH_3	OH	OCH_3	H
**93**	OCH_3	OCH_3	H	H	H	H
**94**	OCH_3	OCH_3	CH_3	H	H	H
**95**	OCH_2O		CH_3	H	H	H
**104**	OCH_3	OCH_3	CH_3	OCH_3	OCH_3	H
**110**	OCH_2O		CH_3	H	H	H

No.	R_1	R_2	R_3
**117**	CH_3	OH	H
**118**	C_2H_5	OH	H
**118**	CH_3	H	H
**120**	CH_3	OH	acetyl
**121**	CH_3	H	acetyl
**122**	C_2H_5	OH	acetyl

Figure S2. The structures of alkaloids extracted from natural sources using ILs or IL solutions.

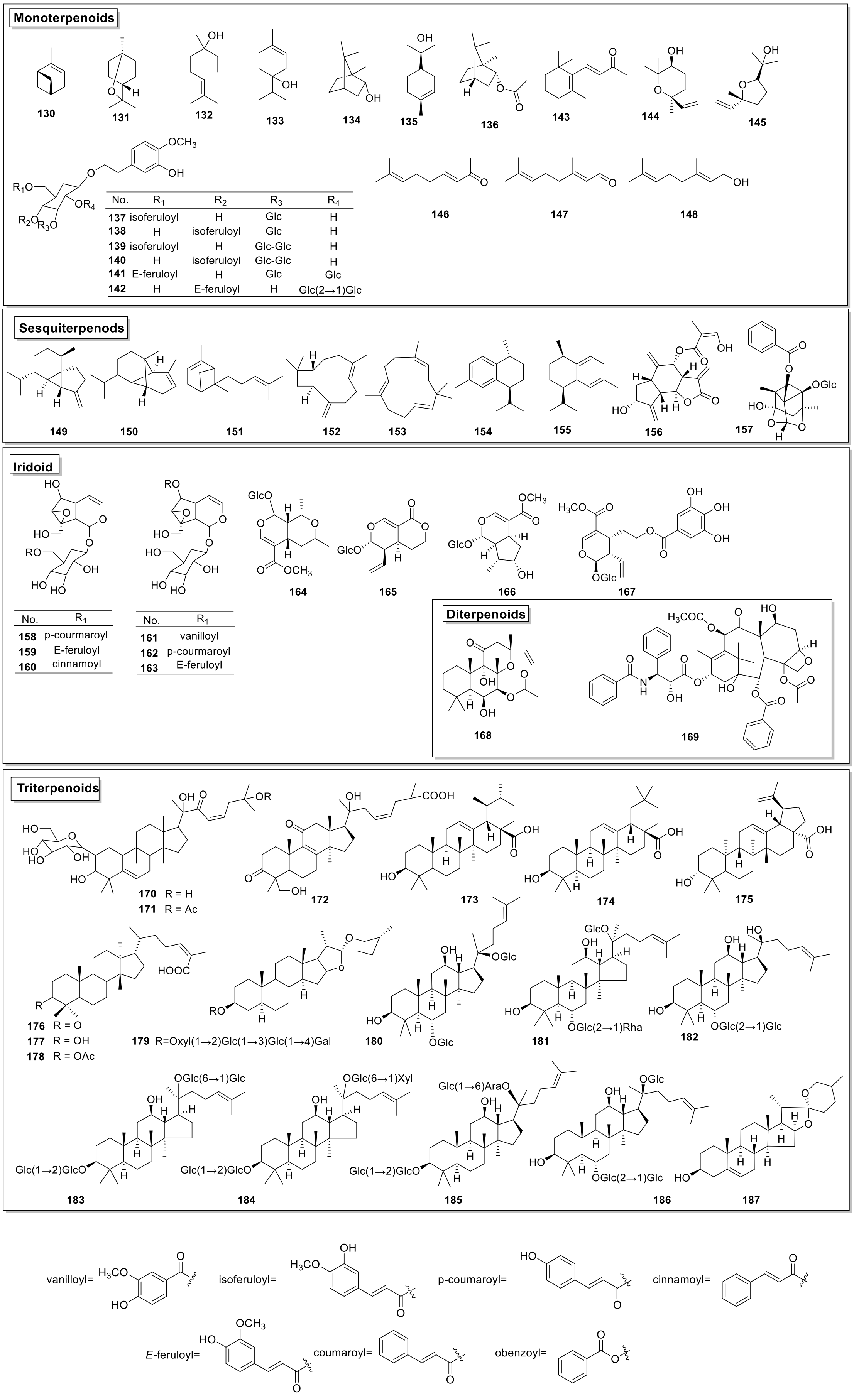

Figure S3 The structures of terpenoids extracted from natural sources using ILs or IL solutions.

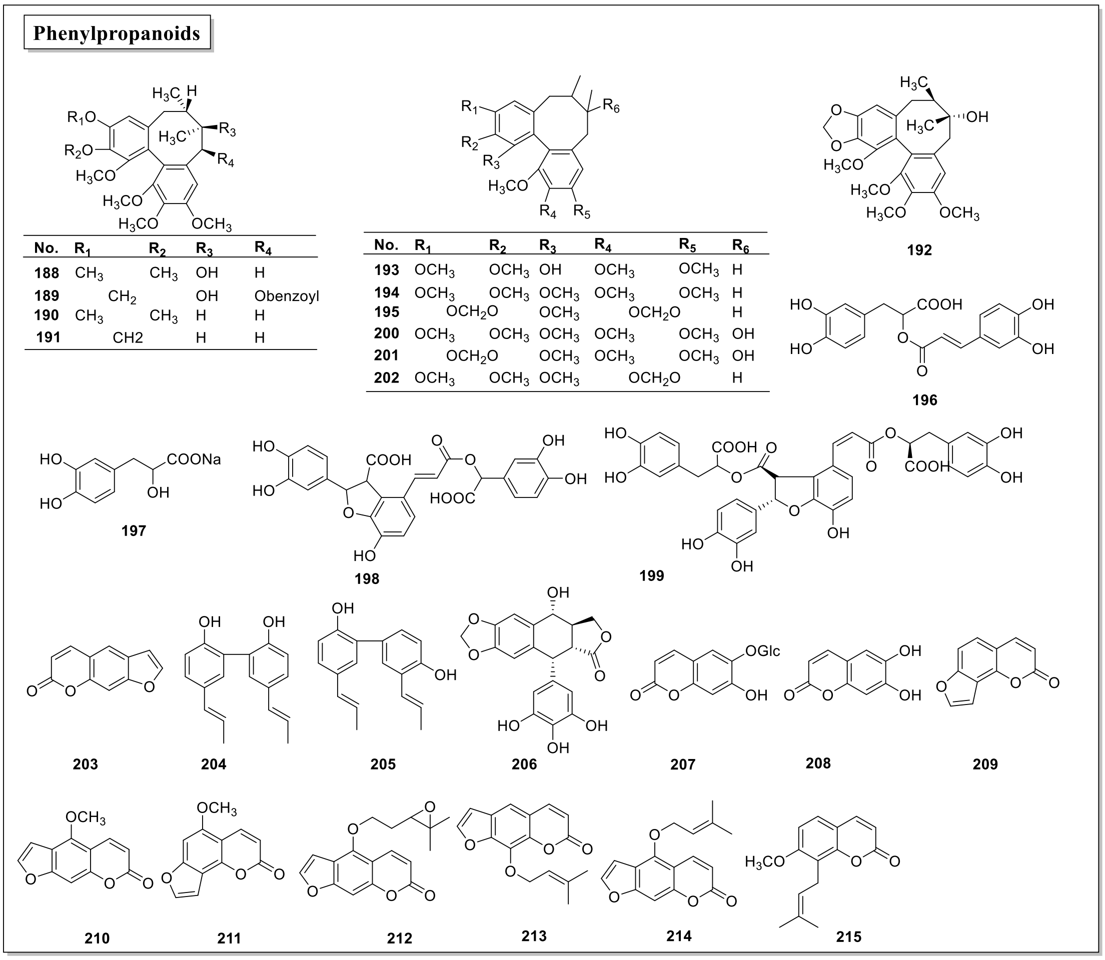

No.	R_1	R_2	R_3	R_4
188	CH_3	CH_3	OH	H
189	CH_2		OH	Obenzoyl
190	CH_3	CH_3	H	H
191	CH2		H	H

No.	R_1	R_2	R_3	R_4	R_5	R_6
193	OCH_3	OCH_3	OH	OCH_3	OCH_3	H
194	OCH_3	OCH_3	OCH_3	OCH_3	OCH_3	H
195	OCH_2O		OCH_3	OCH_2O		H
200	OCH_3	OCH_3	OCH_3	OCH_3	OCH_3	OH
201	OCH_2O		OCH_3	OCH_3	OCH_3	OH
202	OCH_3	OCH_3	OCH_3	OCH_2O		H

Figure S4. The structures of phenylpropanoids extracted from natural sources using ILs or IL solutions.

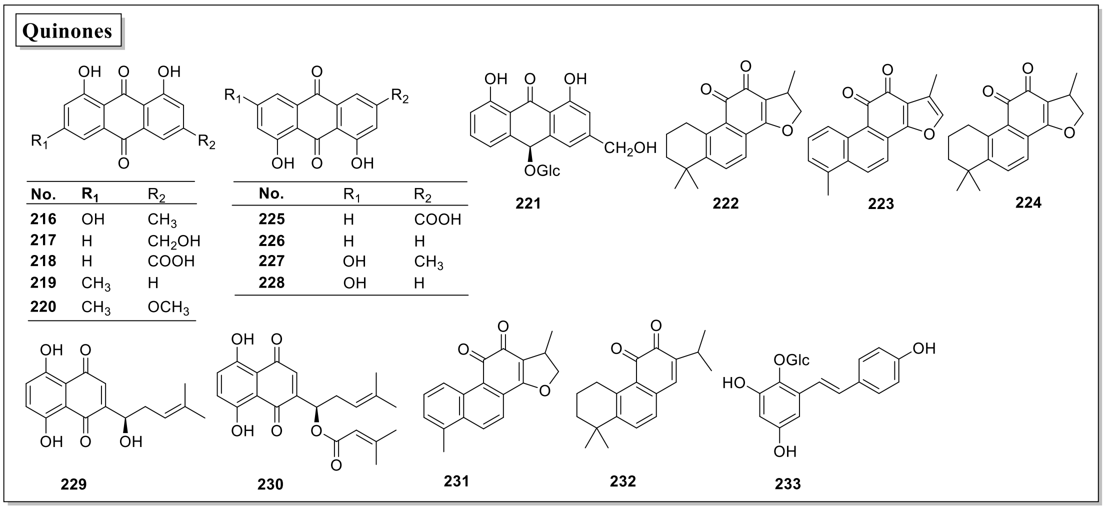

No.	R_1	R_2
216	OH	CH_3
217	H	CH_2OH
218	H	COOH
219	CH_3	H
220	CH_3	OCH_3

No.	R_1	R_2
225	H	COOH
226	H	H
227	OH	CH_3
228	OH	H

Figure S5. The structures of quinones extracted from natural sources using ILs or IL solutions.

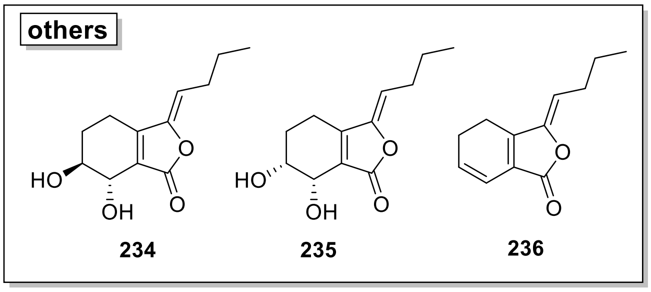

Figure S6. The structures of other compounds extracted from natural sources using ILs or IL solutions.
